# Extracorporeal Membrane Oxygenation as Circulatory Support in Adult Patients with Septic Shock: A Systematic Review

**DOI:** 10.2478/jccm-2024-0017

**Published:** 2024-04-30

**Authors:** Muhammad Faisal Khan, Mohsin Nazir, Muhammad Khuzzaim Khan, Raj Kumar Rajendram, Faisal Shamim

**Affiliations:** Department of Anesthesiology, Aga Khan University Hospital, Karachi, Pakistan; Department of Internal Medicine, Dow University of Health Sciences, Karachi, Pakistan; College of Medicine, King Saud bin Abdulaziz University for Health Sciences, Riyadh, Saudi Arabia

**Keywords:** critical care, sepsis, septic shock, ECMO, systematic review

## Abstract

**Introduction:**

The utilization of extracorporeal membrane oxygenation (ECMO) in adult patients experiencing septic shock is a subject of ongoing debate within the medical community. This study aims to comprehensively address this issue through a systematic review conducted in accordance with the PRISMA guidelines.

**Aim of Study:**

The primary objective of this study is to assess the outcomes of ECMO utilization in adult patients diagnosed with septic shock, thereby providing insights into the potential benefits and uncertainties associated with this treatment modality.

**Materials and Methods:**

Our research encompassed a thorough search across electronic databases for relevant English-language articles published up until April 2023. The inclusion criteria were based on studies reporting on ECMO usage in adult patients with septic shock. Among the eligible studies meeting these criteria, a total of eleven were included in our analysis, involving a cohort of 512 patients. The mean age of the participants was 53.4 years, with 67.38% being male.

**Results:**

In the pooled analysis, the mean survival rate following ECMO treatment was found to vary significantly across different ECMO modalities. Patients receiving venovenous-ECMO (VV-ECMO) and veno-venous-arterial ECMO (VVA-ECMO) demonstrated higher survival rates (44.5% and 44.4%, respectively) compared to those receiving venoarterial-ECMO (VA-ECMO) at 25% (p<0.05). A chi-square test of independence indicated that the type of ECMO was a significant predictor of survival (χ^2^(2) = 6.63, p=0.036). Additionally, patients with septic shock stemming from respiratory failure demonstrated survival rates ranging from 39% to 70%. Predictors of mortality were identified as older age and the necessity for cardiopulmonary resuscitation (CPR).

**Conclusions:**

In septic shock patients, ECMO outcomes align with established indications like respiratory and cardiogenic shock. VV-ECMO and VVA-ECMO suggest better prognoses, though the optimal mode remains uncertain. Patient selection should weigh age and CPR need. Further research is vital to determine ECMO's best approach for this population.

## Introduction

Extracorporeal Life Support (ECLS) is a term that encompasses the use of external mechanical devices for compensating or replacing acute and life-threatening cardiovascular or respiratory insufficiencies or replacing the lung and heart function. One of the modalities of ECLS is extracorporeal membrane oxygenation (ECMO), which utilizes an extracorporeal circuit with a magnetically driven centrifugal rotation pump. In this technique, blood is extracted from a central vein and delivered to an oxygenator, where it undergoes gas exchange and heating, and then returned to a central vein (veno-venous; VV) or artery (veno-arterial; VA), or both (venovenous-arterial; VVA) [1].

The use of ECMO in adults is growing rapidly, and its effectiveness in treating severe respiratory and cardiogenic shock has been well-established [2]. Despite limited evidence, ECMO is currently recommended as rescue therapy for severe respiratory failure and cardiogenic shock in patients who have suffered acute myocardial infarction [3]. Moreover, VA-ECMO is not only indicated in cardiogenic shock caused by myocardial infarction, it can be used also in many other types of cardiac failure, postcardiac surgery or even in cardiac electrical storm [4].

Septic shock is a common, life-threatening condition with high morbidity and mortality rates. The role of ECMO in adult patients with septic shock remains controversial, although several reports have shown favorable outcomes in newborns and children with refractory septic shock [5]. The American College of Critical Care Medicine recommends considering ECMO for the treatment of septic shock in children and neonates, suggesting a potential efficacy of ECMO in adult patients with septic shock [6]. However, due to limited data and guidance, appropriate decision-making in this complex situation is challenging. This systematic review comprehensively evaluates the outcomes of ECMO utilization in adult patients with septic shock, a condition that has been underrepresented in previous studies. By pooling data from eleven studies involving 512 patients, we were able to assess the impact of ECMO mode (VV-ECMO, VVA-ECMO, or VA-ECMO) and patient characteristics (age, gender, comorbidities, etc.) on survival and other clinical outcomes. Our findings provide valuable information for clinicians and patients who are considering ECMO as a potential treatment option for septic shock.

## Materials and Methods

### Study Registration and Methodology

This systematic review was conducted in accordance with the Preferred Reporting Items for Systematic Reviews and Meta-Analyses (PRISMA) guidelines. Institutional review board approval was not required as the study only used data from published literature and did not involve direct human participation.

### Literature Search

Six databases, including PubMed, SCOPUS, CINAHL, ProQuest, Google Scholar, and the Cochrane Library, were searched for English-language articles published from inception until April 2023 using the following keywords and MeSH terms: “sepsis or septic shock or severe sepsis” and “extracorporeal membrane oxygenation or ECMO or extracorporeal life support or extra-corporeal membrane.” The reference lists of relevant studies were also reviewed to identify additional literature, and clinical trial registries, including ClinicalTrials.gov, the WHO International Clinical Trials Registry, and the EU Clinical Trials Registry, were searched for gray literature. Further details of the search strategy are included in the supplementary materials.

### Eligibility Criteria and Study Selection

Inclusion criteria for this systematic review were as follows: randomized controlled trials and observational studies (cohort, case-control, and cross-sectional) and retrospective analyses reporting the use of extracorporeal membrane oxygenation for severe sepsis and septic shock in adults. Non-English language publications were excluded. Participants were adult patients over 18 years of age, of any race and gender, admitted to an intensive care unit with severe sepsis and septic shock. Studies reporting the use of ECMO for any indication other than severe sepsis and septic shock were excluded. Outcome measures of interest included ICU mortality, length of ICU and hospital stay, tissue oxygenation, vasopressor and inotrope requirements, hospital mortality (death from any cause occurring in the hospital after the start of the ECMO) and weaning from ECMO.

### Data Extraction and Quality Assessment

Three reviewers independently evaluated the literature identified from the six databases and manual searches. Titles and abstracts were initially screened, followed by full-text articles that met the inclusion criteria. Disagreements were resolved by a fourth reviewer. Data were extracted independently by three authors using a standardized form, including demographics, study methodology, informed consent, ethics review committee approval, intervention groups, interventions, and results. Reasons for study exclusion were also documented. Risk of bias was assessed using the Scottish Intercollegiate Guidelines Network (SIGN) critical appraisal checklist, with each author independently assessing bias according to standardized descriptions for each type of bias in observational studies (i.e., selection bias, confounder control, performance bias, recall bias, attrition bias, and selective reporting bias).

### Data Synthesis

A narrative synthesis of the reported data was performed to provide an analytical picture of the presentation, management, and outcomes of using ECMO in sepsis and septic shock. Data clustering was performed to identify specific groups of data points for further analysis, followed by concept mapping to identify potential relationships, concepts, and explanations between the clustered data points. Concept mapping allowed for the conceptualization of the efficacy of ECMO for improved outcomes in sepsis and septic shock.

## Results

### Search results and study characteristics

**Fig. 1.**
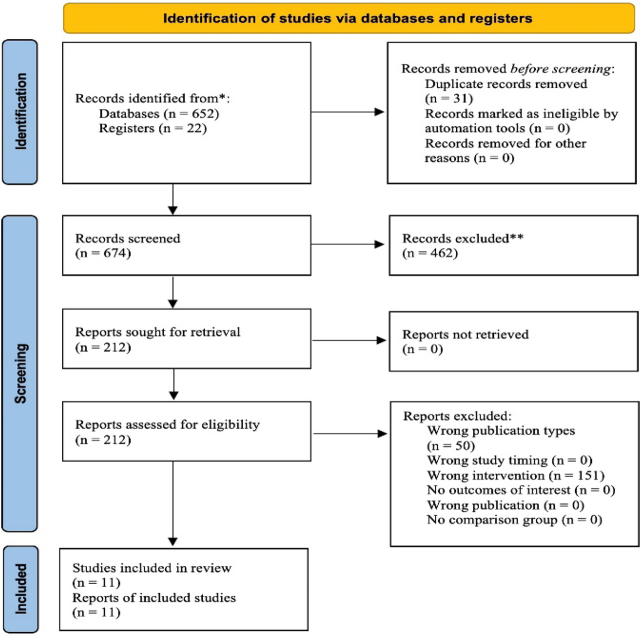
PRISMA flow diagram

Identification and selection of studies were conducted through a comprehensive literature search of computerized databases and registers. A total of 652 studies were identified, with an additional 15 studies from the registers. After removing duplicates and conducting an initial review of titles, 462 studies were excluded. Eight studies were reviewed in full text, with two studies being excluded. Finally, eleven studies [7,8,9,10,11,12,13,14,15,16,17] were selected for qualitative review, as shown in Figure 1 of the PRISMA flow diagram.

Eleven observational studies that investigated the use of ECMO in septic patients were included in the present systematic review. Five of the studies were retrospective cross-sectional studies of the use of VA-ECMO, while two were retrospective cohort studies that included patients treated with VA-ECMO and VV-AECMO, respectively, in patients with septic shock. One study was a propensity-matched analysis of an ECMO registry that included patients treated with VV and VA-ECMO. The remaining five studies were retrospective cohorts. The majority of patients included in these studies were adults with refractory septic shock despite adequate intravascular volume and high-dose infusions of catecholamines and vasopressin.

The inclusion criteria for individual studies varied but generally included patients with sepsis and refractory circulatory or respiratory failure. Exclusion criteria included patients who had previously received ECMO or who were primarily receiving ECMO for respiratory support. The main and associated outcomes of the included studies are summarized in Table 1, which includes a total of 512 patients who received ECMO for septic shock in critical care settings.

**Table 1.** Characteristics of studies included in the systematic review.

**Study No.**	**Study name**	**Study type**	**ICU Mortality**	**Length of ICU stay**	**Length of hospital stay**	**Improvement in tissue oxygenation**	**Vasopressor requirement**	**Survival to the ICU / hospital discharge**	**Other Outcomes**
01.	Park et al., 2014.	32 patients (21 males) with refractory septic shock were retrospectively reviewed. Baseline: Shock-ECMO interval 30.5 hours, CPR duration (median) 23 minutes.	CPR strongly predicts in-hospital mortality after ECMO.	Median 11.1 days (IQR 4.0–26.0). Survivors had longer stay (32.5 days) than non-survivors (7.6 days) with P=0.02.	Not reported	Survivors had lower peak lactate levels (4.5 mmol/l) and higher peak troponin I values (32.8 ng/ml) than non-survivors.	Not reported	7 survived, 19 died from shock/multiorgan failure.	Successful weaning: 40.6% in all patients. Stroke: 3.1% in all patients, none in survivors.
02.	Huang et al.	Retrospective study, 52 patients; inclusion criteria: age >18 years, V-A ECMO, positive culture/serology, exclusion criteria: ECMO primarily for respiratory support.	Not reported	Median 90.1h (IQR 28.3 – 314.7)	Median: 114.1 hours (IQ 52.3 – 404.7)	Not reported	Not reported	Survival: 15% survived, 64% died. Age <60 better survival (P=0.029).	Duration of ECMO (n=52) 15.0 hours IQ (6.1– 29.3). Bleeding at the cannulation site & GI bleeding= 8
03.	Cheng et al. 2013.	Propensity-matched ECMO study: 108 septic vs. 108 non-septic patients, age 16+, VA & VV-ECMO, non-first time ECMO excluded.	Septic ECMO patients had higher mortality, especially in patients over 55 years old.	Not reported	Not reported	Not reported	IABP during ECMO: septic (n=108) 19.0%, non-septic (n=108) 25.3% (P=0.285)	Survival to discharge: 28.7% in septic vs 37.0% without sepsis Survived beyond ECMO: 44.4% in septic vs 56.5% in non-septic patients.	CPR during ECMO is more common in non-septic group (n=108). Post ECMO neurologic deficit higher in non-survivors (n=71).
04.	Brechot et al. 2013	Survey of 14 patients with refractory cardiovascular failure associated with sepsis and other criteria for VA-ECMO use, excluding certain conditions.	ICU mortality in 14 patients was 29%, with 4 deaths during ICU stay (2 during ECMO).	Shorter in non-survivors (median 10 days) compared to survivors (median 17.5 days).	Not reported	ICU patients had a peak troponin value post-ECMO of 5.8 ng/ml.	Not reported	10 (71%) out of 14 patients.	ECMO duration: median (range) (n=10) 5.5 (2–12) days in survivors vs (n=4) 3 (1–7) days in non-survivors.
05.	Banjas et al., 2018	Inclusion: ECMO-treated patients.	56% 6-year hospital mortality.	Mean: 20 (8–31)	Mean hospital stay: 27 (15–40)	Baseline lactate levels: 13 (8–26). PaO_2_/FiO_2_ ratios: 145 (99–233)	Not reported	Not reported	Not reported
06.	Yeo et al., 2017	Patients with septic shock and ARDS were included. (n=8)	Overall survival rate: 50% Successful weaning rate: 62.5%	Not reported	Not reported	Baseline: -Median MAP: 40 mmHg (IQR 33–46) -Median arterial lactate: 7.8 mmol/L (IQR 6.3–16.3)	Not reported	Not reported	Not reported
07.	Lee et al. 2018	Patients: 24 patients (M:F ratio: 6:2). Inclusion: Patients 18 years or older who received ECMO for sepsis.	6 patients died, only 2 were discharged from the hospital.	Median 4 days (1–13)	Not reported	Immediately before the start of ECMO, the median serum lactate level, CRP, and total bilirubin were higher in the survivor group.	Not reported	Survival to discharge: 25% (2 out of 8 patients). 3 patients weaned successfully, but 1 died before discharge	Survival group: Shock to ECMO duration= 25 hours (7–43) Non-survival group: Shock to ECMO duration= 6 hours (1–75)
08.	Ro et al. 2018	Adults (>20 y/o) with refractory shocks who received venoarterial ECMO support.	In-hospital mortality: 93%. 90-day mortality rates: 87.3%.	Not reported	Not reported	Non-survivors had higher arterial lactate, lower platelet count, and higher total bilirubin.	Not reported	Septic shock: 5 patients (7%) survived to discharge	11 patients (15.5%) successfully weaned off ECMO in median 7.9 days.
09.	Han et al. 2016	Inclusion: Patients with persistent circulatory failure or worsened refractory septic shock. Exclude: Patients with advanced malignant tumors, or irreversible neuropathy.	15/23 patients died; 3 died after weaning.	ICU stay was shorter for the survival group (median of 12 days) compared to the death group (median of 16.5 days).	Survival group: Hospital stay = 19 days (range, 17.5–21). Death group: Hospital stay = 16.5 days (range, 13.0– 21.0)	Mean lactate levels were lower in the survival group (4.4 mmol/L) than in the death group (6.8 mmol/L).	Not reported	5 discharged alive, 15 unsuccessful weaning, 3 deaths after weaning.	Not reported
10.	Vogel et al. 2018	Retrospective analysis of ECMO database to identify suitable patients, followed by clinical data extraction from electronic medical records.	3 patients (25%) died, 2 from multiorgan failure and 1 from cerebral edema with brain herniation.	Not reported	Not reported	Baseline lactate: mean 5.0 (range 3.85–6.05). PaO_2_: Mean 9.1 (range 6.4–9.8). pH: mean 7.10 (range 7.08–7.22)	5 patients (41.7%) received vasopressin and 2 patients (16.7%) received adrenaline, dobutamine, or milrinone.	9 (75%) survived after VAV ECMO decannulation.	No deaths reported during follow-up (median 6 months).
11.	Falk et al. 2019	Inclusion: ECMO support received by adult patients with septic shock admitted between January 2012 and December 2017 (n=37).	8/37 patients died.	Not reported	Not reported	Venoarterial patients had a higher Pao_2_-to-Fio_2_ ratio, higher lactate levels, and higher ECMO flow than venovenous patients.	71.4% (5/7) of patients survived who experienced CPR before admission.	ECMO survival: 81.1%. Hospital survival: 78.4%. Long-term follow-up survival: 59.5% (median 46.1 months).	Ten (37%) started venovenous ECMO and 27 on venoarterial ECMO. Venovenous-ECMO was associated with higher risk for in-hospital death (50% vs 11%; p=0.011).

### Quality assessment

The quality assessment of the ten observational studies and one case series included in the systematic review is presented in Table 2. Although there were varying degrees of risk of bias and heterogeneity among the studies, the authors decided to combine the data to provide summary statistics.

**Table 2.** Risk of bias

**Authors’ ID & year of publication**	**Study Design**	**A sample representative of the population (Risk of Selection Bias)**	**Evaluation of the outcome (Risk of Performance Bias)**	**Follow-up long enough**	**Follow up complete**	**Conflict of Interest**	**Other Limitations**
Park et al., 2014	Retrospective review /32 patients	Not a true representative sample (high)	Record review (high)	Not applicable.	Not applicable.	None.	Single-Center Study Small sample size A retrospective review of records
Huang et al., 2013	Retrospective cohort study / 52 patients	Not a true representative sample (high)	Record review (high)	Yes.	Yes.	None.	Single-Center Study Low generalizability
Cheng et al., 2013	Propensity-matched analysis of ECMO registry /108 matched	Not a true representative sample (high)	Record review (high)	Yes.	Yes.	None.	Existence of occult confounders Suboptimal selection because of inappropriate comparability
Brechot et al., 2013	Retrospective cross-sectional survey/14 patients	Not a true representative sample (high)	Record review (high)	Not applicable.	Not applicable.	Two of the authors took payments from companies	Single-Center Study Small sample size No controlled group was matched
Banjas et al., 2018	Retrospective Cohort/131 patients	Not a true representative sample (high)	Record review (high)	Yes.	Yes.	None.	Single-center study Overall, 29% missing data Low generalizability
*Yeo et al., 2017	Brief communication	Case series; not representative sample (High)	Record review (high)	Yes.	Yes.	None.	Single Center case series Small sample size No controlled group A retrospective review of records
Lee et al., 2017	Retrospective cohort/24 patients	Not a true representative sample (high)	Record review (high)	Not applicable	Not applicable	None.	Small sample size • No controlled group was matched
Ro et al., 2018	Retrospective review/71 patients	Not a true representative sample (high)	Record review (high)	Not applicable	Not applicable	None.	Existence of occult confounders A retrospective review of records
Han et al., 2019	Retrospective cohort/23 patients	Not a true representative sample (high)	Record review (high)	Not applicable	Not applicable	None.	Existence of occult confounders Single-Center Study
Vogel et al., 2018	Retrospective case series/12 patients	Not a true representative sample (high)	Record review (high)	Yes.	Yes.	None.	Small sample size • No controlled group was matched
Falk et al., 2019	Retrospective cohort/37 patients	Not a true representative sample (high)	Record review (high)	Yes.	Yes.	None.	Small sample size Single-Center study

### Patient Profile

#### Age

Patient age was reported in all included studies. The median age ranged from 50.9 to 62 years, and the mean age ranged from 48 to 52 years. To estimate the pooled mean age, the median and interquartile range were used to calculate the mean and standard deviation for studies that did not report this data. The estimated mean age for the pooled population was 53.4 ± 15.8 years.

#### Gender

Gender information was available for all studies. Among the eleven studies, the percentage of male participants ranged from 50% to 87.5%. The pooled data from all studies showed that 67.38% of the 512 patients were men.

### Incorporation of CPR Prior to Initiation of ECMO Therapy

In three out of the eleven studies, it was clearly documented that some patients had received cardiopulmonary resuscitation (CPR) before the initiation of ECMO [7,9,11]. Park et al., 2015 reported that 14 (21.9%) patients received CPR prior to ECMO and return of spontaneous circulation (ROSC) was achieved in seven (50%) of these patients. However, all seven patients who did not achieve ROSC prior to ECMO died. Huang et al., 2013 and Cheng et al., 2013 also reported the inclusion of patients who had received CPR before ECMO but did not describe their outcomes. It is likely that these patients received VA-ECMO rather than VV-ECMO. Bréchot et al., 2013 excluded patients who received prolonged CPR (>60 minutes) before ECMO but did not report the number of patients who received CPR before ECMO. Yeo et al., 2016 and Banjas et al., 2018 did not report the inclusion of patients who had CPR prior to ECMO.

### Outcomes

#### Use of Vasopressor, Inotrope, and Intra-aortic Balloon Pump with ECMO

Several studies reported the use of vasopressors and inotropes after weaning from ECMO. Vogel et al., 2018 and Bréchot et al., 2013 reported the number of days patients required catecholamine infusions after ECMO weaning [8,15]. The median duration of catecholamine requirement was similar between survivors (n=10) and non-survivors (n=4). Additionally, Cheng et al., 2013 reported the use of intra-aortic balloon pumps (IABP) in a subset of patients receiving ECMO [11]. Of 108 septic patients, 20 (19.0%) required IABP during ECMO. Interestingly, 25 (25.3%) of 108 non-septic patients also required IABP (p = 0.225). Finally, Vogel et al., 2018 described the use of vasopressin in 5 patients and adrenaline in 2 patients.

#### Lactate and ischemia

Lactate and ischemia were reported in several studies including Park et al., 2015; Bréchot et al., 2013; Ro et al., 2018; and Lee et al., 2018 [7,12,13,15]. Peak lactate concentration was reported in Park et al., 2015, where the median lactate concentration after weaning from VA-ECMO was 15 mmol/l (IQR 9.8–19.5 mmol/l) for all patients (n=32). Median lactate was significantly lower in survivors (n=7; 4.5 mmol/l, IQR 3.6–9.5 mmol/l) compared to non-survivors (n=25; 15.1 mmol/l, IQR 10.2–19.9 mmol/l) with a p-value of 0.03. Limb ischemia was observed in 5 (15.6%) of 32 patients in Park et al., 2015, while Bréchot et al., 2013 reported arterial ischemia in one survivor (n=10) and one non-survivor (n=4). Additionally, Ro et al., 2018 and Lee et al., 2018 found that lactate levels were significantly higher in the non-survivors group.

#### Troponin levels

Park et al., 2015 reported on the peak troponin I concentration of patients after weaning from VA-ECMO [7]. The median maximum troponin concentration for all patients (n=32) was 7.1 ng/ml (IQR 1.6–32.8 ng/ml). Survivors (n=7) had a significantly higher median troponin concentration of 32.8 ng/ml (IQR 16.8–91.6 ng/ml) compared to non-survivors (n=25) who had a median of 3.7 ng/ml (IQR 1.3–19.6 ng/ml) (p=0.02). Bréchot et al., 2013 also reported on peak troponin levels during ECMO, finding a median peak troponin concentration of 5.8 ng/ml (range 0.2–185.0 ng/ml) among 14 patients [13].

#### Length of ICU stay

The length of ICU stay was reported in four studies [7,9.^10^,^13^]. Park et al., 2015 found that the median ICU stay for all patients (n=32) was 11.1 days (IQR 4.0–26.0). Survivors (n=7) had a significantly longer ICU stay with a median of 32.5 days (IQR 18.5–44.6) compared to non-survivors (n=25) with a median of 7.6 days (IQR 3.4–17.3) (p=0.02). Huang et al., 2013 reported a median ICU stay of 90.1 hours (IQR 28.3–314.7 hours; 1.18–13.1 days) for 52 patients. Banjas et al. 2018 found a median ICU stay of 20 days (IQR 17–65) in 19 patients. Bréchot et al., 2013 reported a median ICU stay of 17.5 days (range 8–51) for survivors (n=10) and 10 days (range 1–20) for non-survivors (n=4). Lee et al. 2018 found a median ICU stay of 4 days (range 1–13 days), while Han et al., 2016 reported a median ICU stay of 11 days (range, 8.5 to 17.5).

### ICU mortality

Nine studies reported ICU mortality in patients on ECMO [7,8,9,10,11,12,13,14,15]. Park et al., 2015 found that CPR was an independent predictor of in-hospital mortality after ECMO in multivariate analysis (model 1) [adjusted risk ratio (HR) 4.61 (1.55–13.69) p=0.006]. In model 2, adding the duration of CPR and baseline SOFA score to model 1, higher post-ECMO in-hospital mortality was predicted by increasing the duration of CPR and the higher baseline SOFA score. Cheng et al., 2013 reported that septic patients on VA-ECMO had a higher risk of hospital mortality than non-septic patients [HR 2.54 (95% CI 1.75–3.70) p=0.001]. Mortality was also higher in patients over 55 years [HR 1.56 (95% CI 1.08–2.24), p=0.017]. After excluding patients on VV-ECMO, multivariate analysis found that preexisting sepsis was an independent predictor of mortality in adults requiring VA-ECMO (HR 2.38 p=0.001).

Bréchot et al., 2013 found that out of 14 patients, 4 (29%) died in the ICU (2 during ECMO, 2 deaths in the ICU after weaning from ECMO). Cheng et al., 2013 also reported survival beyond ECMO, with 48 (44.4%) septic patients and 61 (56.5%) non-septic patients surviving beyond ECMO out of 108 patients. Other studies reported ICU mortality without providing additional details, such as Huang et al., 2013, Banjas et al., 2018, Ro et al., 2018, Lee et al., 2018, Han et al., 2016, and Cho et al., 2016.

#### Length of hospital stay

The length of hospital stay was described in three studies [7,9,10]. Huang et al. 2013 reported a median length of stay of 114.1 hours (4.75 days) with an interquartile range (IQR) of 52.3–404.7 hours (2.18–16.9 days). Banjas et al. 2018 reported a median hospital stay of 26 days with an IQR of 18–67 days. In a third study, Park et al. 2015 reported a median hospital stay of 28.3 days (IQR 11.4–45.4 days) for all patients, with a longer median hospital stay for survivors (44.6 days, IQR 32.5–51.5 days) compared to non-survivors (13.4 days, IQR 7.6–23.1 days) (p<0.001).

#### Survival to hospital discharge

Survival to hospital discharge was reported in all included studies [7,8,9,10,11,12,13,14,15,16,17]. The proportion of patients who survived to hospital discharge varied widely between studies. Park et al., 2015 reported that 7 (21.9%) of 32 patients survived hospital discharge. Huang et al., 2013 reported that 8 (15%) of 52 patients survived hospital discharge. Cheng et al., 2013 reported that 31 (28.7%) of 108 patients with sepsis survived beyond discharge. Bréchot et al., 2013 reported that 10 (71%) of 14 patients survived hospital discharge. Yeo et al., 2016 reported an overall survival rate of 50% (i.e., 4 of 8 patients survived). Banjas et al., 2018 reported that 8 (42%) of 19 patients survived hospital discharge. Pooled data from all studies reporting survival to hospital discharge indicated that 147 (28.7%) of 512 patients survived to hospital discharge.

#### Impact of ECMO Type on Hospital Discharge Survival

Survival to hospital discharge was analyzed based on the type of ECMO received in the studies included in this systematic review. Three types of ECMO (VV, VA, and VVA) were used. Park et al., 2015, Huang et al., 2013, and Bréchot et al., 2013 only included patients who received VA-ECMO. Huang et al., 2013 specifically excluded patients who received ECMO for respiratory support (i.e., VV-ECMO). However, Cheng et al., 2013 included patients who received both VV-ECMO and VA-ECMO. Cheng et al., 2013 reported that 10 (45.5%) of 22 patients who received VV-ECMO survived, while 21 (24.4%) of 86 patients who received VA-ECMO survived. Yeo et al., 2016 and Banjas et al., 2018 included only patients who received VVA-ECMO.

Pooled data showed that 10 (45.5%) of 22 patients who received VV-ECMO or arteriovenous CO_2_ removal survived to hospital discharge, 25 (25.5%) of 98 patients who received VA-ECMO survived, and 12 (44.4%) of 27 patients who received VVA-ECMO survived. Compared to VA-ECMO, the use of either VV-ECMO or VVA-ECMO was associated with significantly better survival (χ^2^ 4.15; p=0.042 for both). However, the differences in survival between the various types of ECMO did not reach statistical significance if the data in the VA-ECMO subgroup reported by Cheng et al., 2013 were removed from the analysis. Neither the use of VV-ECMO / arteriovenous CO_2_ removal (χ^2^ 3.46; p=0.063) nor VVA-ECMO (χ^2^ 3.64; p=0.056) was associated with significantly better survival than VA-ECMO.

It is important to note that the data presented by Cheng et al., 2013 as VV- and VA-ECMO are heterogeneous. Some patients in the VV-ECMO subgroup described by Cheng et al., 2013 received arteriovenous CO_2_ removal, which was included in the VV-ECMO subgroup in this review. Some patients in the VA-ECMO subgroup described by Cheng et al., 2013 received VVA-ECMO and others transitioned from VV to VA-ECMO, which was included in the VA-ECMO subgroup in the analysis of pooled data.

## Discussion

The use of extracorporeal membrane oxygenation (ECMO) in adults with septic shock is a topic of debate in the medical community. Despite limited data availability, a systematic review was conducted to define the outcomes of ECMO in this population. The review included eleven studies with a total of 512 septic shock patients who received ECMO. The survival rates varied widely, ranging from 15–71%. However, the data showed that the survival rate of patients who received ECMO for septic shock was comparable to that of patients with other indications for ECMO.

The results of our analysis showed that the survival rate was better in venovenous ECMO (VV-ECMO) and venovenous-arterial ECMO (VVA-ECMO) than in venoarterial ECMO (VA-ECMO). This was expected, as VV-ECMO and VVA-ECMO are indicated for patients with one organ failure only, namely respiratory failure, without cardiac failure. In contrast, VA-ECMO is indicated for patients with both cardiac and respiratory failure. The outcomes associated with VV-ECMO were generally better than those with VA-ECMO, as the circuit is more physiological and patients require only respiratory support. Therefore, VA-ECMO was reserved for cases of septic shock that failed or were likely to fail VV-ECMO. However, the cohort that received VVA-ECMO was arguably even more compromised than those who received VA-ECMO. The differences in outcome between VVA-ECMO and VA-ECMO were influenced not only by sepsis, but also by the location of arterial cannulation - if it was axillary, it usually replaced better the cardiac function; if it was femoral, and the left ventricle was severely affected, it could alter even more the cardiac function. Therefore, in these cases, it was mandatory to introduce another cannula in the axillary artery - if VA-ECMO in these cases were not changed quickly to VVA-ECMO, the prognosis would be altered. But if the left ventricular contractility was not affected, it was enough to have a femoralfemoral VA-ECMO [18].

It is important to consider that case selection may have biased these outcomes. Older age was independently associated with mortality, and some studies suggested that age over 60 years could be considered a contraindication to the use of VA-ECMO to treat septic shock. The requirement for cardiopulmonary resuscitation (CPR) was also an independent predictor of mortality after ECMO. The best results with VA-ECMO were reported in a cohort with a mean age of 48 and patients who received prolonged CPR were excluded.

In some studies, patients who received VV-ECMO had better outcomes than those who received VA-ECMO. However, some patients in the VV-ECMO subgroup received arteriovenous CO_2_ removal rather than ECMO. Thus, the case selection of these studies may have biased outcomes in favor of VV-ECMO. Similarly, the case selection of studies describing the use of VVA-ECMO may have biased outcomes in favor of this modality [19–20].

When VA-ECMO has been used successfully in adults with septic shock, the contribution of myocardial failure has been prominent. Myocardial dysfunction with sepsis is well described, and the use of VA-ECMO for this in adults is rare [21–22]. Furthermore, acute septic cardiomyopathy can suddenly occur in patients with septic shock, and, if recognized early, these patients might benefit from early VA ECMO cannulation. Septic shock that is predominantly vasoplegic with impaired microcirculation (rather than cardiogenic) is not improved by providing microcirculatory hemodynamic support with VA-ECMO [23–24].

This study has limitations as it only includes a small number of observational studies, with a high risk of selection bias due to non-randomized allocation of interventions. Additionally, there is no standard protocol defining the indication for ECMO in patients with septic shock, and the data quality is low despite possible significant benefits suggested by statistical analysis. Further investigation is needed to define the true benefit of VV-, VA-, and VVA-ECMO in patients with refractory sepsis.

The systematic review found that ECMO for septic shock is comparable to ECMO for other indications, and VVA-ECMO may have superior outcomes compared to VA-ECMO in septic shock patients with refractory cardiogenic shock. However, due to ethical concerns, a randomized trial may not be feasible. Therefore, treatment with VVA-ECMO should be considered in septic shock patients with cardiac or respiratory failure, while VV-ECMO may be used in patients with respiratory failure. Conversion to VVA-ECMO should be considered if cardiogenic shock develops later.

## Conclusion

The outcomes of ECMO utilization in septic shock patients exhibit diverse patterns. Vasopressor, inotrope, and IABP use after ECMO weaning varied among studies. Lactate levels and ischemia influenced patient prognosis, with survivors showing lower lactate and instances of ischemia reported. Troponin levels indicated potential prognostic value, with survivors often exhibiting higher concentrations. ICU and hospital stays differed between survivor and non-survivor groups. Predictors of mortality included age, preexisting sepsis, CPR need, and the cause of sepsis (if it was multidrug-resistant or involved other comorbidities). Hospital discharge survival rates varied based on ECMO type, particularly VV-ECMO and VA-ECMO. However, heterogeneity in ECMO reporting warrants cautious interpretation. Overall, this systematic review underscores the complexity of ECMO's impact on septic shock outcomes, necessitating further research for refined treatment strategies. Moreover, other variables that may affect mortality include the timing and the reason of introducing ECMO, the dose of inotropes and vasopressors, and the presence of adverse reactions.
